# Machine learning for predicting intrahospital mortality in ST-elevation myocardial infarction patients with type 2 diabetes mellitus

**DOI:** 10.1186/s12872-023-03626-9

**Published:** 2023-11-27

**Authors:** Panke Chen, Bine Wang, Li Zhao, Shuai Ma, Yanping Wang, Yunyue Zhu, Xin Zeng, Zhixun Bai, Bei Shi

**Affiliations:** 1 Department of Cardiology, Affiliated Hospital of Zunyi Medical University, Zunyi, China; 2 Department of the Clinical Institute, Zunyi Medical University, Zunyi, China; 3 Organ Transplant Center, Affiliated Hospital of Zunyi Medical University, Zunyi, China

**Keywords:** ST-segment elevation myocardial infarction, Diabetes mellitus, Machine learning, Mortality

## Abstract

In an era of increasing need for precision medicine, machine learning has shown promise in making accurate acute myocardial infarction outcome predictions. The accurate assessment of high-risk patients is a crucial component of clinical practice. Type 2 diabetes mellitus (T2DM) complicates ST-segment elevation myocardial infarction (STEMI), and currently, there is no practical method for predicting or monitoring patient prognosis. The objective of the study was to compare the ability of machine learning models to predict in-hospital mortality among STEMI patients with T2DM. We compared six machine learning models, including random forest (RF), CatBoost classifier (CatBoost), naive Bayes (NB), extreme gradient boosting (XGBoost), gradient boosting classifier (GBC), and logistic regression (LR), with the Global Registry of Acute Coronary Events (GRACE) risk score. From January 2016 to January 2020, we enrolled patients aged > 18 years with STEMI and T2DM at the Affiliated Hospital of Zunyi Medical University. Overall, 438 patients were enrolled in the study [median age, 62 years; male, 312 (71%); death, 42 (9.5%]). All patients underwent emergency percutaneous coronary intervention (PCI), and 306 patients with STEMI who underwent PCI were enrolled as the training cohort. Six machine learning algorithms were used to establish the best-fit risk model. An additional 132 patients were recruited as a test cohort to validate the model. The ability of the GRACE score and six algorithm models to predict in-hospital mortality was evaluated. Seven models, including the GRACE risk model, showed an area under the curve (AUC) between 0.73 and 0.91. Among all models, with an accuracy of 0.93, AUC of 0.92, precision of 0.79, and F1 value of 0.57, the CatBoost model demonstrated the best predictive performance. A machine learning algorithm, such as the CatBoost model, may prove clinically beneficial and assist clinicians in tailoring precise management of STEMI patients and predicting in-hospital mortality complicated by T2DM.

**Supplementary Information:**

The online version contains supplementary material available at 10.1186/s12872-023-03626-9.

## Introduction

ST-segment elevation myocardial infarction (STEMI) is a severe type of acute myocardial infarction (AMI) with a poor prognosis and an association with high morbidity and mortality [1–3]. There are multiple risk factors for STEMI, including tobacco use, dyslipidemia, hypertension, diabetes mellitus (DM), and familial history of coronary artery disease (CAD) [4–6]. Particularly, STEMI patients with type 2 diabetes mellitus (T2DM) face an increased risk of cardiovascular complications, with a myocardial infarction rate 2–4 times higher than that in non-diabetic patients [7]. Although recent studies have shown that proactive management of T2DM significantly reduces cardiovascular complications and mortality, the overall prognosis for STEMI patients with T2DM remains poor [8–11].

Traditional models, such as the GRACE score based on clinical data and the TIMI score based on coronary angiography information, have been used to predict patient outcomes [12, 13]. However, the performance of these scores in prognosis prediction has certain limitations, and there is a risk of delayed scoring. Machine Learning (ML), in contrast, demonstrates superior predictive power in identifying interaction patterns between variables, especially in predicting in-hospital mortality and short-term outcomes for acute myocardial infarction patients, compared to conventional statistical methods [14, 15].

Currently, there is no ML model capable of detecting in-hospital mortality for STEMI patients with T2DM. Therefore, this study aims to develop an accurate and effective ML model to predict outcomes for STEMI patients with T2DM, offering better treatment options and reducing perioperative complications in these patients.

## Methods

### Study population

Between January 2016 and June 2020, patients from the affiliated hospital of Zunyi Medical University were recruited [5]. All patients met the diagnostic criteria for STEMI and underwent primary PCI according to the current guidelines [16]. Throughout this study, all procedures involving human participants were in accordance with the Declaration of Helsinki. The present study was approved by the Ethical Evaluation Committee of Zunyi Medical Hospital (ZMU〔2022〕1-177).

### Definitions and data collection

Data about demographics, clinical outcomes, and procedural characteristics were collected using a standardized form. Baseline data on patient characteristics were collected from both medical records and standardized in-person interviews during the index admission for AMI. In this study, T2DM was defined as a chart diagnosis of diabetes or the use of glucose-lowering medication at AMI presentation [17]. Delay was defined as the upper limit of time from onset to the hospital first medical contact > 12 h. The cohort studies were reported in accordance with Transparent Reporting of a Multivariable Prediction Model for Individual Prognosis or Diagnosis (TRIPOD). The electronic health records were used to collect and analyze the complete case data. In our study, the assessment of predictive factors was performed without the knowledge of the participants’ outcomes. An independent observer recorded in-hospital deaths.

### ML algorithm methods

We developed six ML algorithms to model our data: random forest (RF), CatBoost classifier (CatBoost), Naive Bayes (NB), Logistic Regression (LR), gradient boosting classifier (GBC), and extreme gradient boosting (XGBoost). Using a randomization process, we split our dataset into two groups: a training set (70%) to develop the ML models and a validation set (30%) to examine model performance. Using 10-fold cross-validation to ensure the robustness of validation set results.

### Missing data

Complete case data were collected from the electronic health records (EHRs) and analyzed. To simplify the review and ensure accuracy, variables with more than 20% of observations missing were also removed.

### Statistical analysis

Continuous variables are presented as median (IQR), and categorical variables are presented as n (%). During training, the ML-based models were tuned to avoid overfitting, and the models were internally validated using all data via 10-fold cross-validation. The following indicators were used to define model performance: area under the curve (AUC), recall, precision, and F1 value. In the ROC analysis of the entire dataset, a 95% confidence interval was used to assess statistical significance and compare models. All statistical analyses were conducted using Python (version 3.7) and R (version 4.0.2).

## Results

**Fig. 1:**
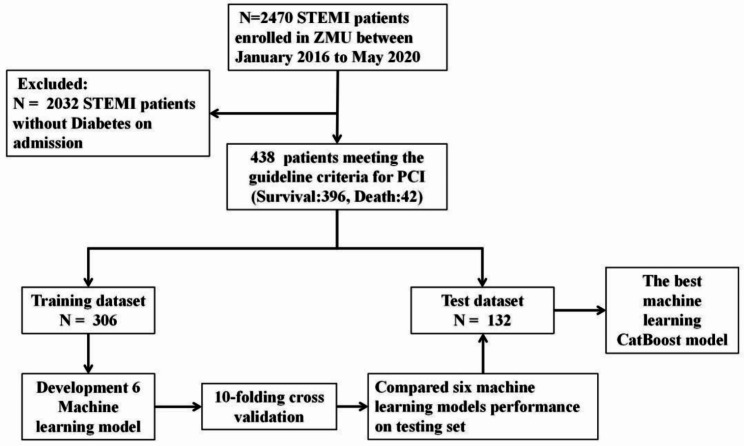
Flow diagram outlining the study process

A total of 438 patients with STEMI registered in the database between January 2016 and January 2020 were included (Fig. 1). The median patient age was 62 (52–71) years, 71% were male, and 42(9.5%)patients died in the hospital. All patients underwent emergency PCI. A comparison of the demographic data and baseline characteristics between patients is shown in Table 1. Six ML models (LR, RF, CatBoost, XGBoost, GBC, and NB) were developed to predict in-hospital mortality rates based on all available features. A comparison of the predictive performances of the six ML algorithm models in the validation set is presented in Table 2. CatBoost (AUC = 0.92 [95%CI:0.909–0.922]),XGBoost(AUC = 0.88[95%CI:0.875–0.891]),RF(AUC = 0.89[95%CI:0.883–0.904]),GBC(AUC = 0.91[95%CI:0.903–0.916]),NB(AUC = 0.87[95%CI:0.859–0.878]),and LR(AUC = 0.84[95%CI:0.833–0.855]) provided similar accuracy values in our study. The GRACE risk assessment tool (AUC = 0.83[95%CI:0.789–0.862]) also demonstrated good discriminatory ability. We investigated the possibility of ensembling and combining different models to further optimize the models, including bagging, boosting, and stacker methods, and found that the performance of the ML models did not improve significantly.

**Fig. 2:**
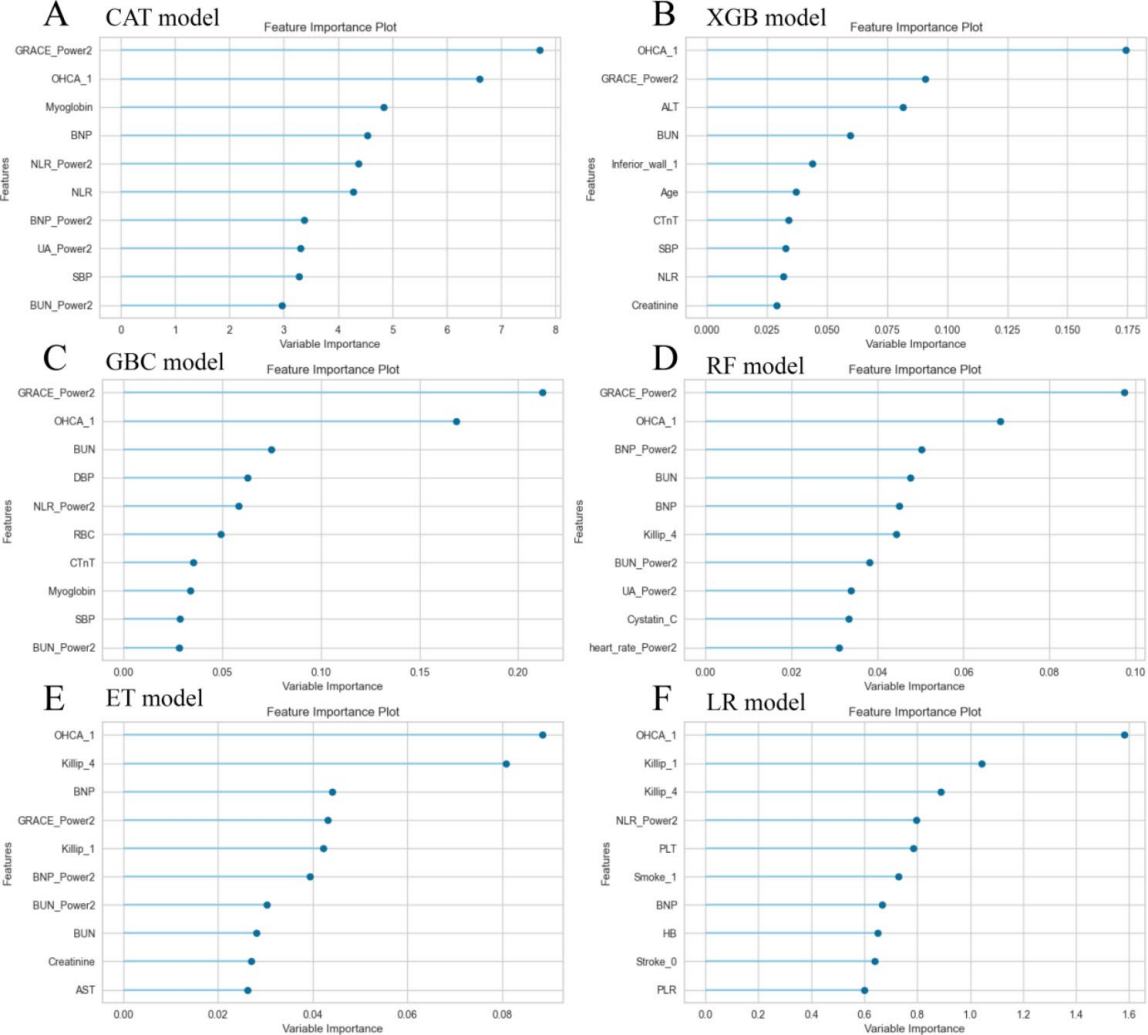
The relative importance of variants in machine learning algorithms

**Table 1:** Demographics and clinical characteristics of patients with and without mortality in the cohort

Variables	Total (n = 438)	Death (n = 42)	Survival (n = 396)	P	statistic
**Demographic characteristics**					
Sex, n (%)				0.021	5.293
female	126 (29)	19 (45)	107 (27)		
male	312 (71)	23 (55)	289 (73)		
Age, Median (Q1,Q3)	62 (52, 71)	71 (62.2, 76.8)	60 (52, 70)	< 0.001	11,548
Weekend on admission, n (%)	159 (36)	12 (29)	147 (37)	0.354	0.859
**Vascular risk factors**					
Hypertension, n (%)	273 (62)	24 (57)	249 (63)	0.574	0.316
Smoke, n (%)	249 (57)	18 (43)	231 (58)	0.078	3.103
Stroke, n (%)	28 (6)	4 (10)	24 (6)	0.33	Fisher
CKD, n (%)	58 (13)	12 (29)	46 (12)	0.004	8.083
Delay, n (%)	126 (29)	11 (26)	115 (29)	0.835	0.044
**Electrocardiographic diagnosis**					
Inferior wall STEMI, n (%)	191 (44)	12 (29)	179 (45)	0.057	3.621
Anterior wall STEMI, n (%)	230 (53)	26 (62)	204 (52)	0.263	1.253
Other STEMI, n (%)	12 (3)	1 (2)	11 (3)	1	Fisher
Right ventricular STEMI, n (%)	7 (2)	1 (2)	6 (2)	0.509	Fisher
**Clinical data**					
Heart rate, Median (Q1,Q3)	81.5 (73, 95)	90.5 (75, 111)	81 (73, 93)	0.018	10166.5
SBP, Median (Q1,Q3)	130 (113, 142)	128 (105.2, 139.8)	130 (114, 142)	0.24	7399
DBP, Mean ± SD	81 ± 16.3	75.9 ± 19.2	81.5 ± 15.9	0.073	-1.831
Shock index, Median (Q1,Q3)	0.6 (0.6, 0.7)	0.7 (0.6, 0.8)	0.6 (0.5, 0.7)	0.037	9940
Killip, n (%)				< 0.001	Fisher
1	349 (80)	16 (38)	333 (84)		
2	47 (11)	5 (12)	42 (11)		
3	19 (4)	6 (14)	13 (3)		
4	23 (5)	15 (36)	8 (2)		
OHCA, n (%)	22 (5)	17 (40)	5 (1)	< 0.001	Fisher
**Laboratory examinations on admission**					
WBC, Median (Q1,Q3)	10.4 (8.5, 13.4)	12.3 (9.5, 18.7)	10.4 (8.3, 13.1)	0.001	10,789
Neutrophil count, Median (Q1,Q3)	8.2 (6, 10.9)	10.1 (8.1, 15.3)	8 (5.9, 10.6)	< 0.001	11360.5
NLR, Median (Q1,Q3)	5.9 (3.3, 9.5)	8.6 (5.8, 13.1)	5.8 (3.1, 9.3)	< 0.001	11,351
PLR, Median (Q1,Q3)	143.6 (95.1, 204.3)	181.2 (95.5, 276)	142.1 (95.6, 195.6)	0.013	10,205
MLR, Median (Q1,Q3)	0.5 (0.3, 0.7)	0.6 (0.4, 0.9)	0.5 (0.3, 0.7)	0.022	10058.5
SIRI, Median (Q1,Q3)	3.8 (2.2, 6.7)	5.6 (3.6, 9.9)	3.6 (2.2, 6.4)	< 0.001	11100.5
SII, Median (Q1,Q3)	1136.5 (635.1, 1968.1)	1823.1 (1243.6, 3138.4)	1078.1 (629, 1879.5)	< 0.001	11,205
HB, Mean ± SD	137.4 ± 21.4	124.7 ± 22.8	138.8 ± 20.8	< 0.001	-3.827
RBC, Median (Q1,Q3)	4.6 (4.1, 5)	4.1 (3.7, 4.8)	4.6 (4.1, 5)	0.002	5856.5
PLT, Median (Q1,Q3)	199 (162, 247)	200.5 (163.5, 274.5)	199 (162.5, 245)	0.593	8711.5
ALT, Median (Q1,Q3)	31 (21, 48)	37 (20, 69)	30.5 (21, 47.2)	0.09	9328
AST, Median (Q1,Q3)	63 (34.5, 134)	128 (47, 241)	59.5 (34, 121.2)	0.01	10048.5
GGT, Median (Q1,Q3)	42 (26, 70.2)	35 (25, 74)	42 (27, 70)	0.846	7867
BUN, Median (Q1,Q3)	5.7 (4.7, 7.6)	8.9 (5.5, 11.6)	5.6 (4.6, 7.3)	< 0.001	11,523
Creatinine, Median (Q1,Q3)	80 (63, 101)	112 (84, 162)	78 (62, 98)	< 0.001	11892.5
UA, Median (Q1,Q3)	361 (287, 435)	449 (368, 545)	355 (280, 419.2)	< 0.001	11,609
CyC, Median (Q1,Q3)	1 (0.8, 1.3)	1.6 (1.1, 1.9)	1 (0.8, 1.2)	< 0.001	11852.5
CK, Median (Q1,Q3)	454 (175, 1153.2)	574.5 (256.5, 1379.2)	423 (174.8, 1105.5)	0.187	9346
CKMB, Median (Q1,Q3)	44 (22, 101)	66 (29.8, 107.8)	43 (21, 101)	0.095	9618
LDH, Median (Q1,Q3)	366.5 (262.2, 581.2)	530 (391, 829.2)	344 (258.8, 561.5)	< 0.001	11,641
HBDH, Median (Q1,Q3)	257 (172, 447.8)	393 (241.5, 631.8)	249.5 (166, 424)	< 0.001	11,338
CTnT, Median (Q1,Q3)	809.8 (175.2, 2413.2)	2294 (648.6, 5431)	746.4 (145.7, 2195.5)	< 0.001	11192.5
myoglobin, Median (Q1,Q3)	233.6 (62.5, 728.4)	705.9 (141.1, 1912)	216.4 (55.8, 659.7)	< 0.001	10,777
BNP, Median (Q1,Q3)	840.9 (236.8, 2616.8)	5483.5 (1445, 11624.5)	753.4 (194.3, 2269.5)	< 0.001	11536.5
Diseased vessel during procedure					
LM, n(%)	16 (4)	1 (2)	15 (4)	1	Fisher
LAD, n(%)	317 (88)	22 (79)	295 (89)	0.119	Fisher
LCX, n(%)	212 (75)	18 (78)	194 (75)	0.916	Fisher
RCA, n(%)	262 (85)	18 (78)	244 (86)	0.354	Fisher
Risk assessment					
GRACE, Median (Q1,Q3)	118 (99, 139)	174.5 (134.2, 198.8)	115 (98, 133.2)	< 0.001	13771.5

Abbreviations: Shock index, ratio of HR to SBP; SIRI, systemic inflammatory response index; SII, systemic inflammatory reaction index; PLR, platelet-to-lymphocyte ratio; NLR, neutrophil-to-lymphocyte ratio; MLR, monocyte-to-lymphocyte ratio; OHCA, out-of-hospital cardiac arrest; GRACE, Global Registry of Acute Coronary Events score; α-HBDH, α-hydroxybutyrate dehydrogenase; BNP, B-type natriuretic peptide; CTnT, cardiac troponin T; CyC, Cystatin C; RCA, right coronary artery; LCX, left circumflex artery; LAD, left anterior descending artery; LM, left main artery; RBC, red blood cell; PLT platelet; AST, Aspartate aminotransferase; ALT, alanine aminotransferase; GGT, gamma-glutamyltransferase; BUN, blood urea nitrogen; UA, uric acid

**Table 2:** Comparison of validation set results of the machine learning models

Model	Accuracy [95%CI]	AUC [95%CI]	Recall [95%CI]	Precision [95%CI]	F1 [95%CI]
CatBoost Classifier	0.93 [0.925–0.932]	0.92 [0.909–0.922]	0.52 [0.485–0.548]	0.79 [0.755–0.815]	0.57 [0.544–0.599]
Gradient Boosting Classifier	0.91 [0.908–0.916]	0.91 [0.903–0.916]	0.46 [0.431–0.486]	0.67 [0.639–0.694]	0.52 [0.493–0.543]
Random Forest Classifier	0.92 [0.918–0.926]	0.89 [0.883–0.904]	0.54[0.511–0.572]	0.74 [0.707–0.766]	0.58 [0.551–0.604]
Extreme Gradient Boosting	0.91 [0.909–0.916]	0.88 [0.875–0.891]	0.52 [0.483–0.55]	0.56 [0.531–0.592]	0.51 [0.478–0.536]
Naive Bayes	0.9 [0.895–0.902]	0.87 [0.859–0.878]	0.73 [0.704–0.746]	0.58 [0.563–0.596]	0.62 [0.609–0.634]
Logistic Regression	0.9 [0.891-0.9]	0.84 [0.833–0.855]	0.4 [0.373–0.427]	0.55 [0.516–0.584]	0.39 [0.36–0.414]
**GRACE**		0.83 [0.789–0.862]	-	-	-
Bagging-Catboost	0.93 [0.925–0.932]	0.92 [0.91–0.923]	0.52 [0.485–0.548]	0.79 [0.755–0.815]	0.57 [0.52–0.622]
Boosting-Catboost	0.92 [0.914–0.923]	0.9 [0.894–0.912]	0.49 [0.461–0.523]	0.68 [0.645–0.705]	0.54 [0.513–0.57]
Blend model-ALL model	0.92 [0.918–0.926]	0.92 [0.91–0.924]	0.52 [0.485–0.548]	0.74 [0.705–0.765]	0.57 [0.537–0.593]
Stacker model-ALL model	0.93 [0.922–0.929]	0.92 [0.918–0.93]	0.48 [0.455–0.512]	0.79 [0.755–0.815]	0.55 [0.526–0.577]

**Fig. 3:**
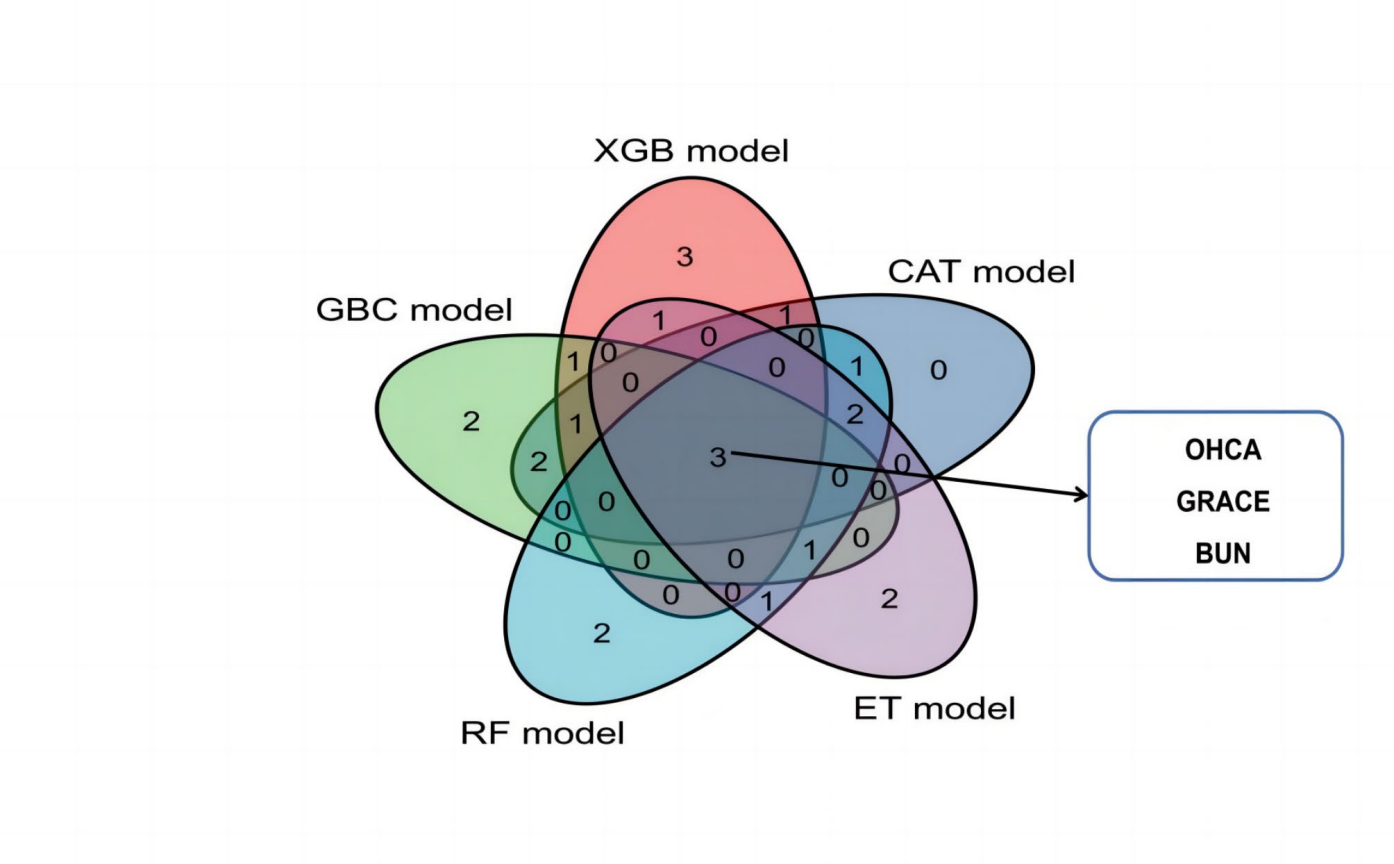
Cross-verification in the top 10 variables of the machine learning models

**Fig. 4:**
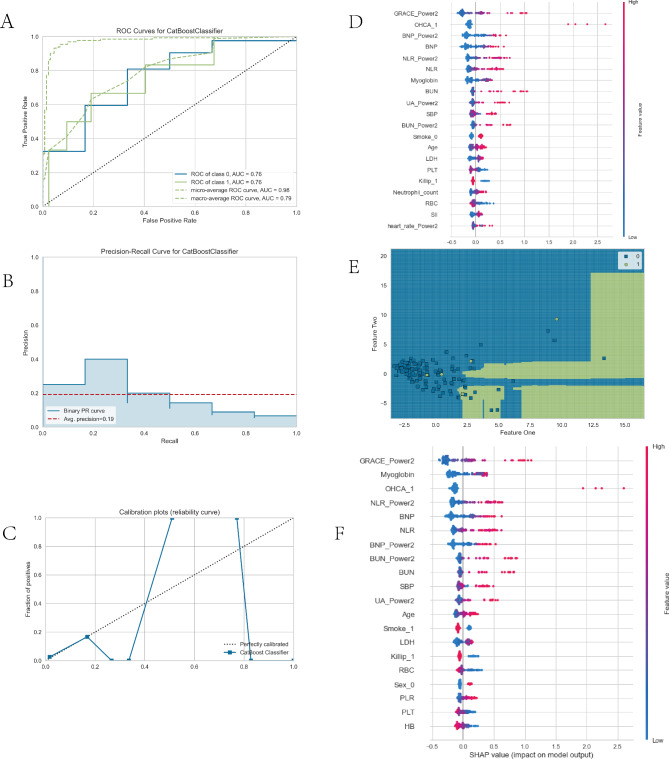
Visualization of CatBoost model performance in the validation set. (**A**) AUCROC curve. (**B**) Precision-recall curve. (**C**) Calibration curve. (**D**) Classification report. (**E**) Decision boundary. (**F**) Feature SHAP value

Figure 2 illustrates the relative importance of the variables in each ML algorithm. Excluding the logistics model, the top 10 most important intersection variables of the other five excellent ML models are shown in Fig. 3. We can see a general trend in the evidence: despite the slight difference in the importance of variables in these ML algorithms, factors including out-of-hospital cardiac arrest (OHCA), GRACE, and blood urea nitrogen (BUN) ranked in the top five (Fig. 3). The neutrophil-to-lymphocyte ratio (NLR), B-type natriuretic peptide (BNP), and cystatin C were ranked in the top ten. Conversely, the cardiac troponin T and creatinine variables contributed little to the prediction. The significance of the high-ranking variables in the CatBoost model decreased in the following order: GRACE, OHCA, myoglobin, BNP, and NLR. There are challenges involved in saving patients who were hypoperfused when they were admitted due to STEMI. The CatBoost model demonstrated the highest performance of the predictive models, with an AUC of 0.92, precision of 0.79, and accuracy of 0.93. Therefore, we selected the CatBoost model as the final predictive model for application to the validation set (Fig. 4).

## Discussion

This study aimed to develop an accurate and user-friendly prediction model for STEMI patients with T2DM to enhance therapeutic decision-making and reduce periprocedural complications. Our approach leveraged machine learning (ML) algorithms, known for their exceptional performance over traditional regression methods in large-scale outcome prediction [14, 15, 18, 19]. We focused on integrating ML techniques with preoperative clinical data for this patient group.

We addressed the challenge of potential data lag in the risk model, which incorporates preoperative, intraoperative, and postoperative clinical data, as this can delay the prediction of adverse events. While logistic regression is often chosen in traditional binary outcome prediction models for its strong predictive power and model interpretability, in our study, CatBoost showed superior performance. Among the five ML models, the GRACE variable and OHCA were the strongest predictors of almost all the analysis methods. Coronary artery disease, particularly acute coronary syndrome, is the leading cause of OHCA in Asians. The survival rate for Asians with OHCA is 3.0% [20]. The treatment of post-resuscitation is dependent on emergency coronary angiography and PCI. Therefore, this study again validated the effectiveness of the GRACE score in a cohort of patients with T2DM and STEMI while finding that ML models had better model discrimination and accuracy. Moreover, it is worth mentioning that, in the case of medical sample imbalance, some studies propose that the model integration technology can significantly improve model performance and capability [14, 21].

ML in clinical medicine faces challenges like data quality and processing complexity, with ML model features often being subtle and difficult for clinicians to interpret. Despite these challenges, ML has achieved significant breakthroughs in biomedicine. For example, ML algorithms have been utilized to analyze complex, high-dimensional data in 12-lead ECGs, predicting artery occlusion in myocardial infarction cases [22]. Liang et al.‘s study effectively used ML to predict heart failure risk during hospitalization for patients with acute anterior wall ST-segment elevation myocardial infarction, employing parameters such as VF, CAP, age, LVEF, and NT-pro-BNP peak levels. This approach enabled the identification of high-risk patients, guiding personalized and proactive management strategies [23]. Further, research by Tofighi et al. demonstrated the efficacy of ML in identifying high-risk STEMI patients for adverse events during follow-up, aiding in crafting individualized treatment plans to improve outcomes and lessen disease burden [24]. Avvisato’s study illustrated ML’s powerful capacity to dissect individual variations in the whole brain functional connectome, aiding in the diagnosis of neurological diseases and predicting clinical outcomes [25]. Those predictive models can be seamlessly integrated into the emergency visit process to aid physicians in determining the prognosis of patients’ conditions. By prioritizing higher-risk patients for more immediate and comprehensive care and monitoring or scheduling follow-ups for lower-risk patients, this approach not only optimizes emergency physicians’ workflow but also ensures that patients receive care tailored to their specific risk profiles. Therefore, understanding the synthetic significance of clinical data samples and the models and methods to accurately diagnose diseases or predict the occurrence of adverse events will remain a persistent focus in medical integration.

## Limitations

This study also has some limitations. Firstly, being a single-center study, the generalizability of our results might be limited. Additionally, its retrospective design raises the possibility of selection bias, which could affect the interpretation of results and the applicability of prediction models. Secondly, like other similar studies, ours relied on clinical data elements such as medical history, physical examination, and lab findings as input features. This reliance potentially restricts the model’s applicability in real-world settings and results in some models lacking interpretability. Thirdly, akin to most studies [26], ours used a single dataset for training and testing the model, but the sample size was insufficient for precise testing and training, mainly due to a lack of adequate positive samples. Even though synthetic sampling techniques were employed, the improvement was not significant, indicating a need for studies with larger sample sizes in this field. Fourthly, the absence of external validation in our study could lead to overfitting. Fifth, While the GRACE score’s inclusion might enhance the model’s predictive accuracy, it can also complicate the interpretation, as the score itself is a composite measure. This layer of abstraction might obscure the individual impact of the clinical variables constituting the GRACE score. Relying heavily on an existing score can affect the robustness of the model, potentially making it more sensitive to any biases or limitations inherent in the GRACE score. Lastly, the indicators included in the analysis were not comprehensive, missing crucial variables such as a timeline of medical visits, medication usage, etc., all of which could influence the outcomes. Therefore, future research should employ larger sample sizes and more scientific methodologies.

## Conclusion

In this study, various ML methods were used to establish in-hospital death prediction models for AMI complicated by diabetes. Compared with the traditional model and GRACE score, the predictive discrimination of the ML model based on the CatBoost algorithm was more accurate. Although this ML algorithm presently has a good prediction ability, it still requires further scientific and reasonable external verification.

### Electronic supplementary material

Below is the link to the electronic supplementary material.

**Supplementary Material 1: **sFig1.ROC curve analysis of the GRACE score and six machine learning models in the overall dataset

**Supplementary Material 2**

## Data Availability

The datasets used and/or analyzed during the current study are available from the corresponding author upon reasonable request.
