# Supplementary figures and images for: Machine learning for predicting intrahospital mortality in ST-elevation myocardial infarction patients with type 2 diabetes mellitus
Source: BMC Cardiovasc Disord. 2023 Nov 27;23:585. doi: 10.1186/s12872-023-03626-9 (PMC10683359; doi:10.1186/s12872-023-03626-9)

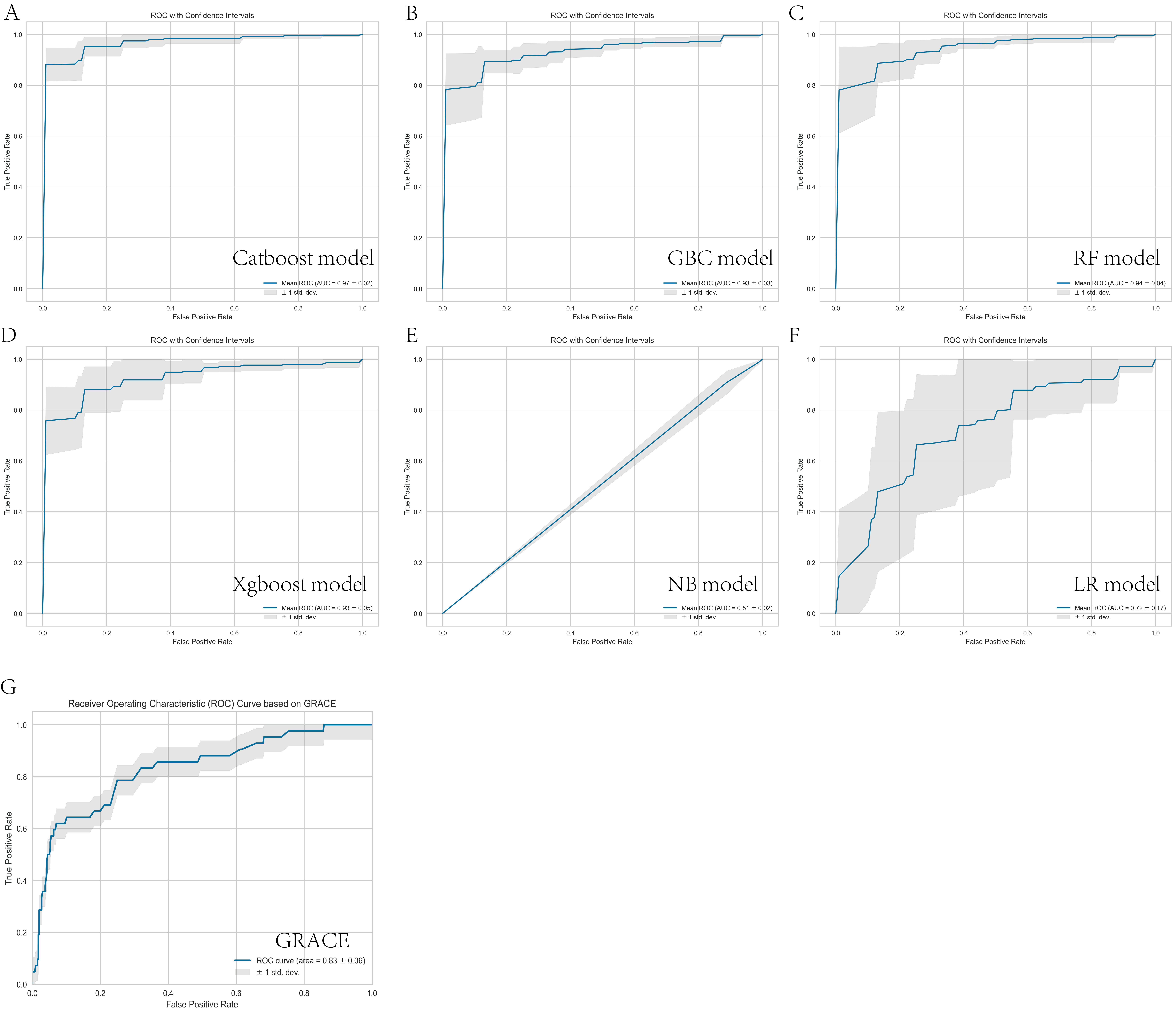

Supplement: Supplementary file 1 — Supplementary Material 1: sFig1.ROC curve analysis of the GRACE score and six machine learning models in the overall dataset [file 12872_2023_3626_MOESM1_ESM.png]
