# Supplementary material for: Machine learning for predicting intrahospital mortality in ST-elevation myocardial infarction patients with type 2 diabetes mellitus
Source: BMC Cardiovasc Disord. 2023 Nov 27;23:585. doi: 10.1186/s12872-023-03626-9 (PMC10683359; doi:10.1186/s12872-023-03626-9)
Supplement: Supplementary file 2 — Supplementary Material 2 [file 12872_2023_3626_MOESM2_ESM.docx]

**sFig1**.ROC curve analysis of the GRACE score and six machine learning models in the overall dataset.(A) ROC curve of Catboost model in overall dataset.(B).ROC curve of GBC model in overall dataset.(C).ROC curve of RF model in overall dataset.(D).ROC curve of Xgboost model in overall dataset.(E).ROC curve of NB model in overall dataset.(F).ROC curve of LR model in overall dataset.(G)ROC curve of GRACE score in overall dataset.
